# Structure of the *N*-glycosidase MilB in complex with hydroxymethyl CMP reveals its Arg23 specifically recognizes the substrate and controls its entry

**DOI:** 10.1093/nar/gku486

**Published:** 2014-06-11

**Authors:** Gong Zhao, Geng Wu, Yan Zhang, Guang Liu, Tiesheng Han, Zixin Deng, Xinyi He

**Affiliations:** State Key Laboratory of Microbial Metabolism and School of Life Sciences and Biotechnology, Shanghai Jiao Tong University, 1954 Huashan Road, Shanghai 200030, China

## Abstract

5-Hydroxymethylcytosine (5hmC) is present in T-even phage and mammalian DNA as well as some nucleoside antibiotics, including mildiomycin and bacimethrin, during whose synthesis 5hmC is produced by the hydrolysis of 5-hydroxymethyl cytidine 5′-monophosphate (hmCMP) by an *N*-glycosidase MilB. Recently, the MilB–CMP complex structure revealed its substrate specificity for CMP over dCMP. However, hmCMP instead of CMP is the preferred substrate for MilB as supported by that its K_M_ for CMP is ∼27-fold higher than that for hmCMP. Here, we determined the crystal structures of MilB and its catalytically inactive E103A mutant in complex with hmCMP. In the structure of the complex, Phe22 and Arg23 are positioned in a cage-like active site resembling the binding pocket for the flipped 5-methylcytosine (5mC) in eukaryotic 5mC-binding proteins. Van der Waals interaction between the benzene ring of Phe22 and the pyrimidine ring of hmCMP stabilizes its binding. Remarkably, upon hmCMP binding, the guanidinium group of Arg23 was bent ∼65° toward hmCMP to recognize its 5-hydroxymethyl group, inducing semi-closure of the cage-like pocket. Mutagenesis studies of Arg23 and bioinformatics analysis demonstrate that the positively charged Arg/Lys at this site is critical for the specific recognition of the 5-hydroxymethyl group of hmCMP.

## INTRODUCTION

5-Hydroxymethyl cytosine (5hmC), also known as the ‘sixth base,’ was discovered in T-even phage and mammalian DNA. This interesting epigenetic modification has drawn worldwide attention recently to the role of this new base as well as the enzymology behind its synthesis and recognition. The deoxycytidylate (dCMP) hydroxymethylase (CH) encoded by the bacterial phage transfers the methylene group from methylene tetrahydrofolate (CH2THF) to the C5 atom of dCMP, and then uses water molecule to hydrate the methylene group to generate hydroxymethyl dCMP (hmdCMP), which is then incorporated into the phage DNA during DNA replication (1). 5hmC in mammalian DNA is produced postreplicatively by the Tet1-catalyzed oxidation of 5-methylated cytosine (5mC) (2). The 5hmC in DNA could be further oxidized by the Tet3 dioxygenase to 5-carboxylcytosine that is specifically recognized and excised by thymine-DNA glycosylase (TDG), and thus Tet3-mediated DNA carboxylation is involved in the epigenetic reprogramming of zygotic paternal DNA following natural fertilization (3).

**Figure 1.**
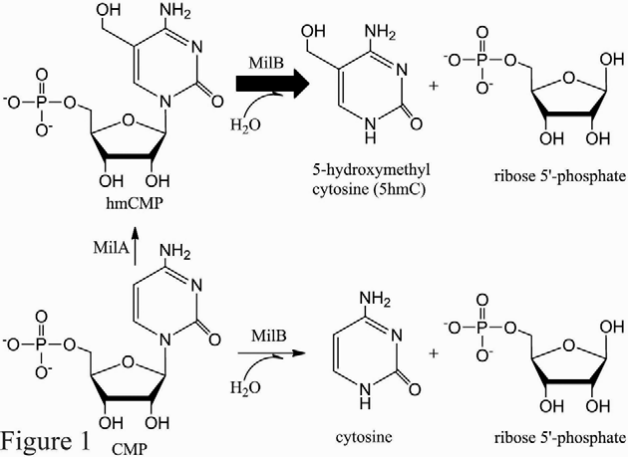
The *N*-glycosidase MilB catalyzes the hydrolysis of hmCMP, its natural and preferred substrate, to 5hmC and ribose 5′-phosphate.

Some natural metabolites and antibiotics, such as bacimethrin (4), 5-hydroxymethyl blasticidin S (5) and mildiomycin (6), also contain 5hmC that is derived from hmCMP. For example, in the mildiomycin biosynthesis pathway of *Streptomyces remofaciens* ZJU5119, MilA catalyzes the hydroxymethylation of CMP, using the same enzymatic mechanism as the enzyme CH from phage (7). 5-Hydroxymethylcytosine is then released via hydrolysis of the *N*-glycosidic bond of hmCMP by MilB. To a less extent, MilB can also directly hydrolyze CMP to ribose 5′-phosphate and cytosine (Figure 1), which is further processed by downstream enzymes in the mildiomycin biosynthesis pathway and is incorporated into the dehydroxymethyl mildiomycin as a minor component of the fermentation products of *S. remofaciens* (8).

Although MilB belongs to the nucleoside 2′-deoxyribosyltransferase (NDT) family, it catalyzes the hydrolysis of hmCMP and CMP rather than hmdCMP or dCMP. Recently, the Steven E. Ealick group reported the crystal structure of MilB in complex with CMP and revealed that the F17Y mutation of MilB completely reversed its substrate specificity from CMP to dCMP (9). However, CMP is not the natural and the most preferred substrate for MilB. The K_M_ value of MilB for CMP (9) is ∼27-fold higher than that for hmCMP (8), showing that the binding affinity of MilB toward hmCMP is ∼27-fold greater than that toward CMP. This observation prompted us to relate the different affinities of MilB for hmCMP and CMP to the discrimination of 5mC from cytosine by some 5mC-binding proteins. Base flipping is a newly discovered mechanism for 5mC recognition, by which 5mC was flipped out of the DNA double helix and recognized by a cage-like binding pocket (10–13).

So far, there has been little structural investigation into the recognition of 5hmC. In this study, we determined the crystal structures of wild-type (WT) MilB by itself to a resolution of 1.8 Å and its catalytically inactive E103A point mutant in complex with its natural substrate hmCMP to a resolution of 2.4 Å. The selectivity of hmCMP by MilB is attributed to the distortion of the guanidinium group of Arg23 induced by hydrogen bonding with the hydroxymethyl group, leading to semi-closure of the cage-like binding pocket where the hmCMP was trapped in. The cage-like pocket is very similar to that in 5mC-binding proteins such as SET and RING-associated (SRA) domains of UHRF1 and the dioxygenase NgTet1 (11,13). Measurements of the enzymatic kinetic constants of WT MilB and its various point mutants at Arg23 support the key role of Arg23 of MilB in the hydroxymethyl group-specific recognition of hmCMP.

## MATERIALS AND METHODS

### Site-directed mutagenesis of MilB

Genes encoding WT MilB and BlsM were cloned into the pET28a (Novagen) vector, with N-terminal 6xHis tags. All mutant plasmids were constructed by the whole-plasmid polymerase chain reaction and DpnI digestion method (14), and verified by sequencing. The plasmids and the primers used in this study are listed in Supplementary information, Table S1 and S2.

### Protein expression and purification

Proteins were all overexpressed in the *Escherichia coli* strain BL21(DE3) at 20°C; 10-ml culture grown overnight from a single colony was inoculated into 1 L of Luria Broth medium supplied with 50-μg/ml kanamycin and 34-μg/ml chloramphenicol. The culture was incubated at 37°C to OD_600_ = 0.6–0.8, and induced by the addition of 0.2-mM Isopropyl β-D-1-thiogalactopyranoside (IPTG) for another 20 h at 16°C. The cells were harvested and resuspended in 20-ml binding buffer (20-mM Tris–HCl, pH 8.0, 20-mM imidazole and 300-mM NaCl), and lysed by sonication in an ice bath. After centrifugation at 16 000 g for 30 min at 4°C, the supernatant was applied to 2-ml Ni-NTA column (Qiagen) pre-equilibrated by the binding buffer. The column was washed by 60-ml binding buffer and 10-ml washing buffer (20-mM Tris–HCl, pH 8.0, 50-mM imidazole and 300-mM NaCl). The column was then eluted with 10-ml elution buffer (20-mM Tris–HCl, pH 8.0, 300-mM imidazole and 300-mM NaCl). All the eluant was collected and further purified by the Superdex200 gel filtration chromatography (GE Healthcare) equilibrated with 10-mM Tris–HCl, pH 8.0, 100-mM NaCl and 2-mM dithiothreitol. The purified proteins were visualized by sodium dodecylsulphate-polyacrylamide gel electrophoresis and Coomassie bluse staining, and the protein concentration was determined by using the Bradford Protein Assay Kit (Bio-Rad). The combined peak fractions were concentrated to 10 mg/ml. Selenomethionine (SeMet)-substituted MilB were expressed using the methionine-autotrophic *E. coli* strain B834 cultured in M9 media and purified similarly, except that 20-mM β-mercaptoethanol was added before sonication.

### Crystallization

Crystallization trials for full-length MilB were performed at 14°C using the hanging-drop vapor-diffusion method in 48-well plates. Typically, 1-μl reservoir solution was mixed with 1-μl protein solution and equilibrated against 1-ml reservoir solution. Initial crystallization screening trials were performed using Crystal Screen, Index, PEG/Ion and SaltRx screen kits from Hampton Research.

After 2 weeks, small crystals of full-length MilB were obtained from condition number 6 of the Crystal Screen kit, which consists of 30% (w/v) polyethylene glycol 4000, 0.2-M magnesium chloride and 0.1-M Tris–HCl, pH 8.5. Longer and thicker crystals were obtained by decreasing the concentration of polyethylene glycol 4000 to 15–20%. After further optimization and microseeding efforts, diffracting crystals were obtained from 16% (w/v) polyethylene glycol 4000, 0.08-M magnesium chloride and 0.1-M Tris–HCl, pH 8.5, using the hanging-drop vapor-diffusion method in 48-well plates at 14°C.

SeMet-MilB was crystallized at 14°C in 16% (w/v) polyethylene glycol 4000, 0.08-M magnesium chloride and 0.1-M Tris–HCl, pH 8.5. The MilB-E103A point mutant protein was crystallized in the same condition. The MilB-E103A/hmCMP complex was obtained by soaking the MilB-E103A crystal in the reservoir solution containing 2-mM hmCMP. The substrate hmCMP was obtained by one-step conversion of CMP by purified MilA, followed by the purification procedure described as reported (15).

Crystal diffraction datasets of SeMet-MilB, MilB and the MilB-E103A/hmCMP complex were collected at the BL17U1 beamline at Shanghai Synchrotron Radiation Facility (SSRF) using an ADSC Quantum 315r CCD area detector, and processed using HKL2000 (16).

Structure determination SeMet-MilB crystals belong to the C2221 space group and contain one molecule in the asymmetric unit. Its structure was determined to 2.25 Å by the singlewavelength anomalous diffraction (SAD) method using PHENIX (17,18).

MilB crystals belong to the C2221 space group, with one molecule in the asymmetric unit. Its structure was determined to 1.8 Å by the molecular replacement method with Phaser (19), using the structure of SeMet-MilB as the searching model. After model-building by Coot (20) and refinement by REFMAC (21), the final model has an R/Rfree of 18.3%/21.6% (Supplementary information, Table S3) and includes MilB residues 12–169.

The MilB-E103A/hmCMP complex crystals belong to the C2221 space group, with one molecule in the asymmetric unit. Its structure was determined to 2.4 Å by the molecular replacement method with Phaser (17), using MilB structure as the searching model. The final model, including MilB residues 12–170 and the bound hmCMP, has an R/Rfree of 19.2%/25.7% (Supplementary information, Table S3).

### Analytical high-performance liquid chromatography

Reaction mixtures were analyzed by reverse-phase high-performance liquid chromatography (HPLC) on Agilent 1200 HPLC system equipped with quaternary pump and thermostat autosampler (25°C). The stationary phase was a Nucleodur 100–5 CN-RP column (15 cm × 4.6 mm, 5-μm particles). The LC eluant (flow rate of 0.6 ml/min) consisted of 2% methanol in water. Chromatograms were detected using the absorbance at 270 nm. Ultraviolet-vis spectra of substrate and product peaks were also collected and assessed.

### Preparation and LC analysis of hmCMP and mCMP for *in vitro* enzymatic assays

5hmCMP was synthesized from CMP in a reaction containing formaldehyde and tetrahydrofolate catalyzed by recombinant CMP hydroxymethylase MilA (8). To make 5mCMP, 5mCTP (TriLink BioTechnologies, Inc.) was reacted at 30°C overnight with Apyrase (New England Biolabs Inc.) to catalyze the removal of the γ-phosphate from adenosinetriphosphate (ATP) and the β-phosphate from ADP. The phosphate from Adenosine monophosphate (AMP) is not removed. Here Apyrase can efficiently convert 5mCTP to 5mCMP as well. After the reaction, the Apyrase was heat inactivated, and the reaction products were used as the substrates to measure the *N*-glycosidic bond hydrolysis activity of the MilB and MilB-R23M.

To separate the 5mCMP from CMP and 5hmCMP, an Agilient Eclipse XDB-C18 column (4.6 × 150 mm, 5-μm particles) was used with conditions as below for the running LC. The LC eluant (flow rate of 0.3 ml/min) consisted of 2% acetonitrile and 98% water (0.1% formic acid). Chromatograms were detected using the absorbance at 270 nm.

### Enzymatic kinetic parameters measurement for MilB and BlsM

Kinetic parameters were monitored on the basis of production of cytosine/5hmC from CMP or hmCMP catalyzed by WT MilB, MilB-R23M and WT BlsM. Each enzyme was incubated with various concentrations of the substrate in 50-mM Tris–HCl, pH 7.2, for 30 min at 37°C, and then the reactions (with a total volume of 10 μl) were quenched by boiling the mixture at 100°C for 10 min. After centrifugation at 18 000 g for 5 min, the samples were analyzed by HPLC as described above. The structures of the reaction products were determined by QTOF/MS (Agilent G6530A). Kinetic parameters were calculated by fitting the enzymatic data to the Michaelis–Menten equation by the non-linear regression analysis (Prism5; GraphPad Software Inc.).

### Normal mode analysis

The crystal structure coordinate of MilB was used for the normal mode analysis conducted using the web server developed by K. Suhre and Y.H. Sanejouand (http://www.igs.cnrs-mrs.fr/elnemo/start.html). Files of the 7th normal mode (i.e. the first vibrational mode with the lowest vibrational frequency, which is the most major vibrational mode of the system under investigation) generated by the server were used for analysis.

### Figure preparation

The figures in this paper were prepared using PyMOL (http://pymol.sourceforge.net), ChemDraw (Cambridge Biosoft), Coreldraw (Corel), Prism5 (GraphPad Software Inc.) or Excel (Microsoft), and compiled in Adobe Photoshop.

### Accession codes

The atomic coordinates and structure factors of MilB (residues 12–169), as well as the MilB (residues 12–170, with the E103A point mutation)/hmCMP complex have been deposited in the Protein Data Bank with accession numbers 4OHR and 4OHB, respectively.

## RESULTS

### Crystal structures of MilB by itself and its catalytically inactive E103A point mutant in complex with hmCMP

To understand the substrate recognition and the enzymatic catalysis mechanism of MilB, we determined its crystal structure independently of the Ealick group (Supplementary information, Table S1). In the crystal, there is one molecule of MilB per asymmetric unit. However, a dimer is related by the crystallographic two-fold symmetry. Our size exclusion chromatography result also confirmed the dimerization state of MilB in solution (Supplemental information, Figure S1), which is consistent with the quaternary structures of both Rcl (22) and purine deoxyribosyltransferase (23), two members belonging to NDT family.

**Figure 2.**
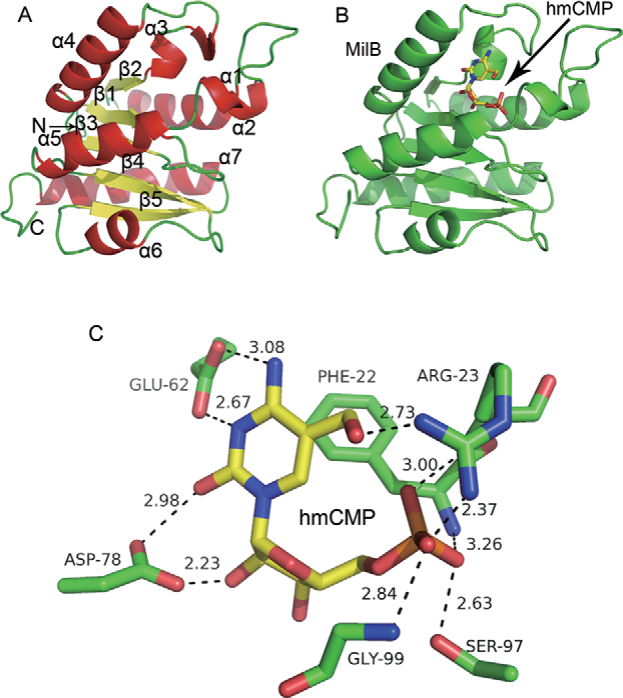
Structures of MilB by itself and its catalytically inactive E103A point mutant in complex with hmCMP. (**A**) The MilB structure adopts an overall α/β-twist fold, with β-strands depicted in yellow and α-helices in red. (**B**) Overall structure of MilB-E103A (colored in green) in complex with hmCMP, whose carbon, nitrogen, oxygen and phosphorus atoms are colored in yellow, blue, red and orange, respectively. (**C**) The close-up view of the hmCMP-binding interface at the active site of MilB.

The final model at 1.8 Å resolution contains residues 12–169 of MilB for each protomer (the full-length protein consists of 170 residues), which possesses an α/β-fold with a five-stranded flavodoxin-like parallel β-sheet with a standard order of 21345. The β-sheet is flanked on both sides by α-helices in an asymmetric manner (Figure 2A).

Based on the previous structural study on NDT, which shows that the conserved glutamic acid is the active-site nucleophile (24), we constructed the E103A point mutant of MilB, which does not possess the hmCMP/CMP hydrolysis activity, and determined the crystal structure of MilB-E103A in complex with hmCMP (Figure 2B; Supplementary information, Table S1). The MilB-E103A/hmCMP complex also crystallizes with one molecule per asymmetric unit. The final model at 2.4 Å resolution contains residues 12–170 of MilB-E103A in complex with one hmCMP molecule. When in complex with hmCMP, MilB has essentially the same structure as that by itself, with a root-mean-square deviation (RMSD) value of 0.223 Å. The hmCMP molecule is located in a deep surface pocket of MilB, surrounded by α-helices α1, α2, α3, α4 and α5 (Figure 2B).

**Figure 3.**
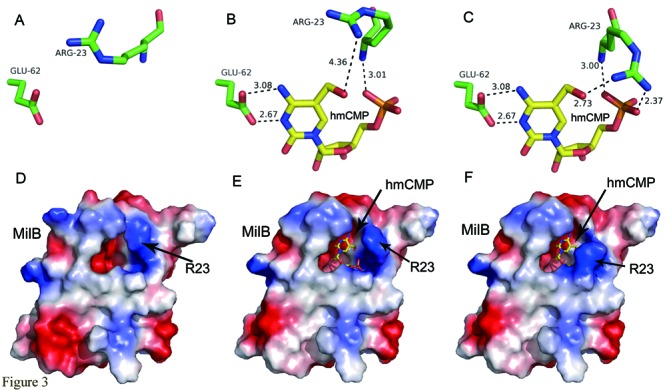
Arg23 at the active site of MilB undergoes a dramatic conformational change upon hmCMP binding. (**A**, **D**) Arg23 adopts an ‘open’ conformation in the hmCMP-unbound MilB structure. (**B**, **E**) The ‘intermediate’ conformation of Arg23 in the MilB−hmCMP complex structure. (**C**, **F**) The ‘closed’ conformation of Arg23 in the MilB−hmCMP complex structure. (D–F) The electrostatic surface potential of MilB, with blue and red representing positively and negatively charged surface areas, respectively.

Our crystal structures of MilB alone and MilB in complex with hmCMP are very similar to that of MilB in complex with CMP (PDB code: 4JEM) reported previously (9), with RMSD values of 0.306 and 0.287 Å for 140 and 141 aligned Cα atoms, respectively (Supplementary information, Figure S2). The most prominent difference among these structures is Arg23. In the structure of MilB alone, Arg23 exists in an extended conformation. In contrast, in the structure of MilB in complex with hmCMP, Arg23 adopts bended conformations and its side chain guanidinium group makes hydrogen bonds with the 5-hydroxymethyl group of hmCMP (Figures 2C and 3). On the other hand, in the structure of MilB in complex with CMP (PDB code: 4JEM), the position of the side chain of Arg23 was not determined and there are no coordinates for the side chain of this residue in the PDB file. Interestingly, in the structure of MilB in complex with CMP, Arg23 resides in a short α-helix, α1 (9). However, in the structure of MilB in complex with hmCMP, this short α-helix melts into a loop (Supplementary information, Figure S2). Other parts of MilB are approximately the same in these three structures.

### Key residues at the active site of MilB for the specific recognition of hmCMP

The structure of the MilB-E103A/hmCMP complex provides details for how MilB specifically recognizes the 5hmC, the ribose and the phosphate moieties of hmCMP (Figure 2C). Six residues of MilB, Phe22, Arg23, Glu62, Asp78, Ser97 and Gly99, play the most important roles in the substrate binding. The benzene ring of Phe22 forms planar stacking interactions with the pyrimidine ring of hmCMP. Additionally, Arg23 hydrogen bonds with the 5-hydroxymethyl group of the 5hmC base, and makes another hydrogen bond to the phosphate group of hmCMP. Besides, Glu62 accepts a hydrogen bond from the 4-amino group of the 5hmC base and, if protonated, would donate a hydrogen bond to the N3 atom of hmCMP. Furthermore, the side chain of Asp78 forms a couple of hydrogen bonds with hmCMP, one with the ribose moiety and the other with the 5hmC moiety (Figure 2C). Apart from these four critical residues, Ser97 and Gly99 are also involved in the hydrogen bonding interactions with the phosphate moiety of hmCMP.

To summarize, Phe22, Arg23, Glu62 and Asp78 interact with the 5hmC moiety, among which only Arg23 is involved in the specific recognition of the hydroxymethyl group of 5hmC; Asp78 contributes to the binding to the ribose moiety, whereas Arg23, Ser97 and Gly99 provide contacts with the phosphate moiety of hmCMP.

### Arg23 undergoes a dramatic conformational change upon hmCMP binding and functions as a gate to control the substrate entry to the active site pocket

When the crystal structure of MilB by itself was compared with that in complex with hmCMP, it was found that Arg23 exists in different conformations in these structures. In the crystal structure of MilB by itself, Arg23 adopts a relaxed and open conformation, with its side chain completely stretched out. Its guanidinium group points outward, facing away from the active site of the hmCMP-binding pocket (Figure 3A and D).

**Figure 4.**
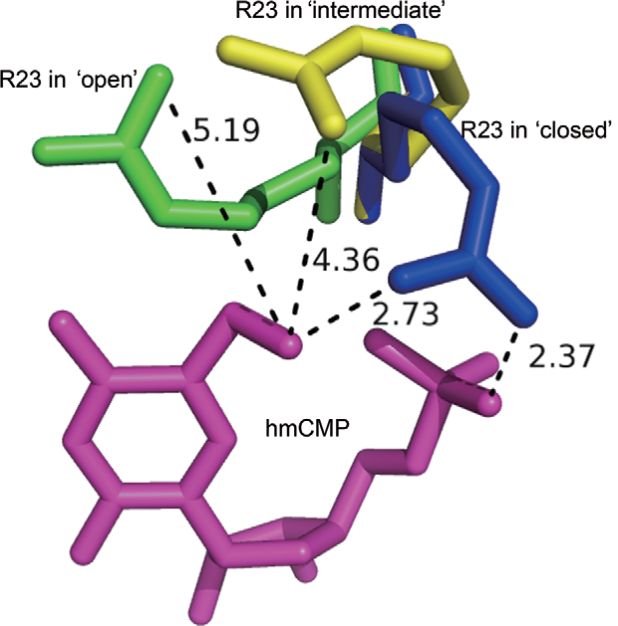
Superimposition and comparison of the different conformations of Arg23 in the hmCMP-bound and -unbound MilB. The conformation of the versatile Arg23 transits from ‘open’ to ‘intermediate’ to ‘closed’ upon hmCMP binding. Arg23 is colored in green, yellow and blue in the ‘open’, ‘intermediate’ and ‘closed’ conformations, respectively.

In the electron density map of the MilB-E103A/hmCMP complex, the side chain of Arg23 can be placed in two alternative ways, suggesting that it varies between two different conformations, ‘intermediate’ and ‘closed’ (Supplemental information, Figure S3). In the ‘intermediate’ state, the side chain of Arg23 employs a half-bended-half-stretched conformation, with its guanidinium group 4.36 Å away from the oxygen atom of the hydroxymethyl group of hmCMP (Figure 3B and E). On the other hand, in the ‘closed’ conformation, Arg23 is completely bended back toward the active site pocket of MilB, so as to recognize hmCMP. Its guanidinium group makes two hydrogen bonds with the hydroxymethyl group and the phosphate group of hmCMP, with distances of 2.73 and 2.37 Å, respectively (Figure 3C and F). Superimposition of different states of MilB further supports the versatility of Arg23, with its conformation transitioning from ‘open’ to ‘intermediate’ to ‘closed’ upon hmCMP binding (Figure 4).

### A positively charged residue corresponding to Arg23 of MilB in its homologs might also be crucial for their specificities for hmCMP over CMP

**Figure 5.**
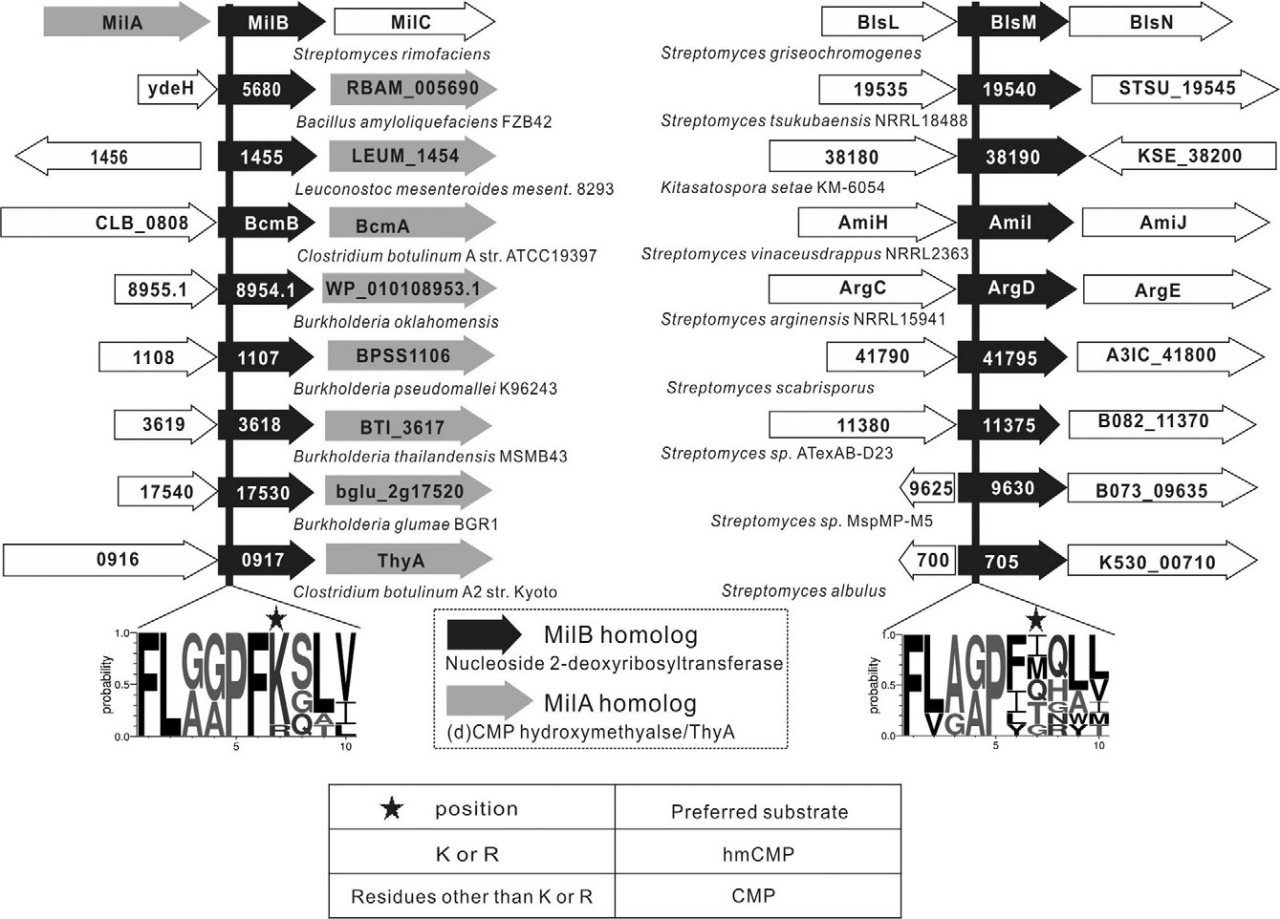
Representations of the genomic organization context of MilB, BlsM and their homologs in several different bacterial species. On the left side, MilB and its homologs containing the hmCMP substrate specificity motif ‘F-L-(G/A)-(G/A)-P-F-(R/K)-X-L-(L/V/T),’ in which the critical ‘R/K’ residue is marked with a black star, are all genetically linked with putative MilA homologs (usually annotated as ThyA or dCMP hydroxymethylase). On the right side, this kind of genomic organization does not exist in BlsM and its homologs, in which the ‘R/K’ position is replaced by various other amino acids such as I/M/Q/T/G (also marked with a black star).

There are 54 close homologs of MilB in the database, with *E* values ≤9e−10. They can be classified into two groups based on whether they are genetically linked with homologs of MilA or not. These putative MilA homologs are annotated either as thymidylate synthase A (ThyA) or CH. ThyA catalyzes the C5 methylation of dUMP to form dTMP, whereas CH firstly methylates dCMP and then hydrates the methyl group to form hmdCMP (Supplementary information, Figure S4). On the other hand, MilA and BcmA catalyze the hydroxymethylation of CMP to form hmCMP (Supplementary information, Figure S3). The methyl donors for the above three types of reactions are all 5,10-methylenetetrahydrofolate (CH2THF). The general outcome of the reactions catalyzed by ThyA/CH/MilA is methylation or hydroxymethylation at the C-5 atom of the pyrimidine ring. Consistent with our structural finding, all the MilB homologs genetically linked with putative ThyA/CH/MilA homologs possess a conserved ‘F-L-(G/A)-(G/A)-P-F-(R/K)-X-L-(L/V/T)’ motif, with a positively charged Arg/Lys residue at the position corresponding to Arg23 of MilB. By contrast, this site in the nine MilB homologs, which are not linked with any ThyA/CH/MilA homolog, is a variable amino acid other than Arg/Lys (Figure 5). This observation strongly supports the key role of Arg23 in the recognition of the hydroxymethyl group of hmCMP.

Given the similar orientations of key residues in the binding pockets for 5mC or 5hmC of their respective recognizing enzymes, the ‘P-F-(R/K)’ motif may be responsible for discriminating 5mC from cytosine. In the structure of NgTet1 in complex with 5-methyl cytosine DNA (13), Phe295 and Arg224 form planar π stacking contacts with the extra-helical 5mC. Like NgTet1, in the structure of MilB in complex with hmCMP, the conserved Phe22 forms planar stacking interactions with the pyrimidine ring of hmCMP, and Arg23 makes hydrogen bonding interactions with the hydroxymethyl and phosphate groups of hmCMP (Supplementary information, Figure S5).

### Hydrolytic activities of WT MilB and its various point mutants at Arg23 toward hmCMP or CMP

**Figure 6.**
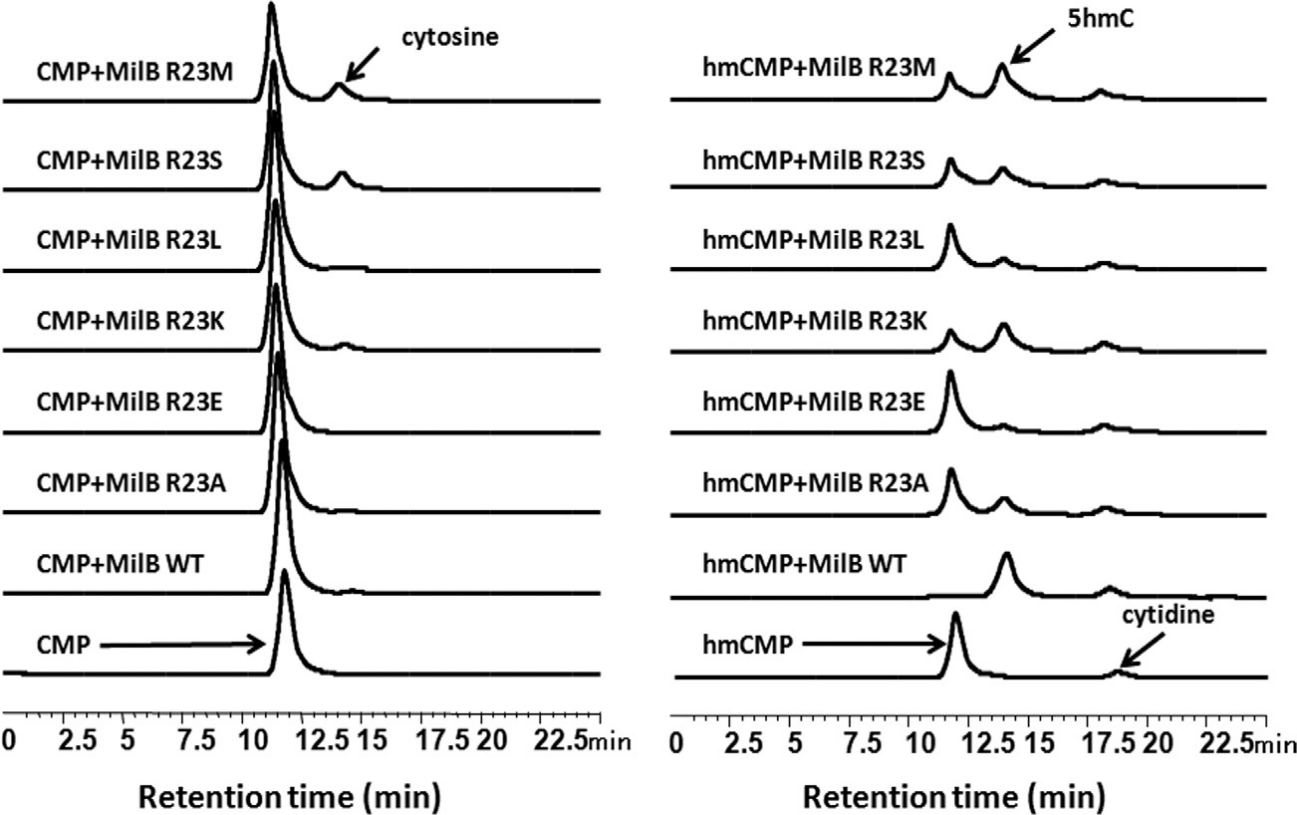
Measurement of the enzymatic activities of WT MilB and various point mutants at Arg23. CMP or hmCMP was incubated with WT MilB, MilB-R23M, -R23S, -R23L, -R23K, -R23E and -R23A, and the production of 5hmC was monitored by the HPLC analysis to measure their *N*-glycosidase activities. MilB-R23E exhibited the lowest activity toward hmCMP, and no cytosine was observed in the reaction with CMP. MilB-R23A and MilB-R23L showed very low activities with hmCMP, and almost no cytosine was produced in the reaction with CMP. MilB-R23K maintained relatively high activity toward hmCMP and CMP. MilB-R23M and MilB-R23S, both having lower activities toward hmCMP than WT MilB, displayed significantly higher activities toward CMP compared to WT MilB.

To verify the substrate recognition mechanism of MilB deduced from our crystal structures, by which Arg23 plays a key role in the discrimination of hmCMP from CMP, point mutants of MilB with single point mutations of R23E, R23K, R23L, R23A, R23M and R23S were constructed and used to measure their hydrolysis efficacy against CMP and hmCMP. The production of cytosine and 5hmC was monitored by the HPLC analysis to measure the *N*-glycosidase activity (Figure 6); the structures of the products were determined by the Q-TOF/MS (Supplementary information, Figure S6, upper and middle). The MilB-R23E mutant exhibited the lowest activity toward hmCMP, which makes sense in that the original charge-stabilized hydrogen bond between the phosphate of hmCMP and the guanidinium group of Arg23 is now changed to a repulsion between the negative charges carried by the phosphate of hmCMP and the glutamate. In contrast, the mutation of R23K in MilB had the smallest effect on the hydrolysis efficiency of hmCMP, possibly due to the compensation of Arg23 by a positively charged lysine at this key site. In addition, when Arg23 was mutated to non-polar amino acids such as alanine or leucine with short side chains, the hydrolysis activities of both mutants were severely impaired to very low levels. Furthermore, when Arg23 was mutated to methionine, which appears at the corresponding position of the CMP-specific *N*-glycosidase BlsM in the blasticidin S biosynthetic pathway, MilB-R23M displayed reduced activity toward hmCMP compared with WT MilB, but its activity toward CMP was significantly enhanced (Figure 6).

Kinetic parameters of MilB for CMP (9) and BlsM for CMP (25) were previously reported (shaded rows in Table 1). In this study, we determined the steady-state kinetic parameters for WT MilB and MilB-R23M using either CMP or hmCMP as the substrate (Table 1, Supplementary information, Figure S7). MilB has a 53-fold binding preference for hmCMP (*K*_M_=71.55 μM) over CMP (*K*_M_=3800 μM), and a 2.68-fold difference in turnover rate between hmCMP (*k*_cat=_53.13 × 10^−3^ s^−1^) and CMP (*k*_cat=_19.83 × 10^−3^ s^−1^); therefore, the strikingly higher hydrolysis efficiency of the hmCMP (*k*_cat_/*K*_M_=742.62 M^−1^ s^−1^) than CMP (*k*_cat_/*K*_M=_5.2 M^−1^s^−1^) by MilB is largely contributed by the binding affinity between MilB and hmCMP. This conclusion is in part reinforced by mutation of Arg23→Met23, which has a 3.39-fold improvement in the binding affinity for CMP (*K*_M=_1122 μM), and a slight decrease for hmCMP (*K*_M_=86.63 μM) by comparison to that for MilB.

**Table 1.** Summary of the enzymatic kinetic constants of WT and the R23M point mutant of MilB

enzyme	substrate	*K*_M_ (μM)	*k*_cat_ (10^-3^ s^-1^)	*k*_cat_/*K*_M_ (M^-1^ s^-1^)
MilB	hmCMP	71.55 (11.66)	53.13 (2.625)	742.62 (126.99)
MilB	CMP	3800.00 (500.00)	19.83 (1.00)	5.20 (0.7)^[9]^
MilB-R23M	hmCMP	86.63 (10.67)	15.61 (0.6)	180.17 (23.05)
MilB-R23M	CMP	1122.00 (100)	30.45 (0.93)	27.14 (2.49)
BlsM	hmCMP	327.50 (66.16)	67.00 (5.60)	204.58 (41.33)
BlsM	CMP	39.00	26.00	675.00^[25]^

**Figure 7.**
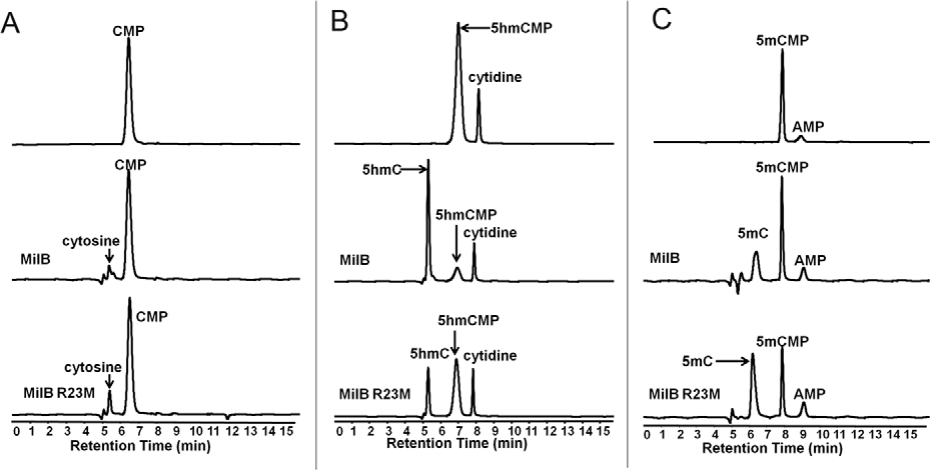
Comparison of catalytic efficiency of MilB and MilB-R23M toward CMP, 5hmCMP and 5mCMP. (**A**) CMP reacted with MilB and MilB-R23M. The product cytosine is indicated and confirmed by the Q-TOF/MS (Supplementary Figure S6). (**B**) 5hmCMP reacted with MilB and MilB-R23M. Target product 5hmC is indicated. The presence of an extra peak was due to co-purification of substrate sample 5hmCMP and cytidine. (**C**) 5mCMP reacted with MilB and MilB-R23M. The product 5mC is indicated. 5mCMP is made from 5mCTP by Apyrase. An additional peak corresponding to AMP generated by the removal of β- and γ-phosphate of ATP, which is provided in the control buffer of Apyrase (NEB). All reactions were incubated at 37°C for 30 min.

According to the structural information, Arg23 is not directly involved in the hydrolysis of the *N*-glycosidic bond, but it is in direct contact with the phosphate group and the hydroxymethyl cytosine. But mutation of Arg23→Met23 caused a decrease of *k*_cat_ for hmCMP (53.61→15.61 M^−1^s^−1^) and an increase of *k_cat_* for CMP (19.83→30.45 M^−1^s^−1^) (Table 1, Supplementary information, Figure S7), indicative of unknown changes in the protein-substrate recognition in addition to alteration of the binding affinity upon the replacement of arginine with the methionine. To minimize the changes of the substrate structure, 5mCMP was prepared (Supplementary information, Figure S7) and measured for 5mC formation (Figure 7C, Supplementary information, Figure S6, bottom) by MilB and MilB-R23M. Compared to 5hmCMP, MilB showed ∼1.8-fold and ∼12.5-fold decreases in the hydrolysis of 5mCMP (Figure 7C, middle) and CMP (Figure 7A, middle), respectively, demonstrating interaction between the polar hydroxyl group and the guanidinium group plays an important role in improving the hydrolytic efficiency of the *N*-glycosidic bond. In the absence of interaction between these two polar groups, MilB-R23M displayed ∼4.3-fold and ∼3.5-fold enhancement in the hydrolysis activity toward 5mCMP (Figure 7C, bottom) and 5hmCMP (Figure 7B, bottom) as compared to CMP, demonstrating that an additional methyl group in the substrate made itself closer to the shortened side chain of methionine in the mutant protein. On the other hand, MilB-R23M showed more efficient hydrolysis activity to CMP (Figure 7A, bottom) and 5mCMP (Figure 7C, bottom) than WT MilB (Figure 7A and C, middle); this observation was attributed to stronger van der Waals force between methionine and methylcytosine/cytosine than that between guanidinium group and methylcytosine/cytosine. Therefore, the positive charge and the side chain length of Arg23 are essential for the discrimination of hmCMP from CMP.

## DISCUSSION

### The critical Arg23 residue on MilB recognizes the hydroxylmethyl moiety and determines its specificity for hmCMP over CMP

In this study, structural analysis of MilB by itself and its E103A mutant in complex with hmCMP revealed the basis of its substrate specificity for hmCMP over CMP. By comparing the structure of MilB by itself with that of MilB-E103A in complex with hmCMP, it is found that Arg23 in the substrate-unbound MilB faces outward from the active site pocket and is about 5 Å away from the supposed binding site of the hydroxymethyl group, forming an ‘open’ conformation (Figure 3A and D). In contrast, in the electron density map of the MilB-E103A/hmCMP complex, the side chain of Arg23 can be placed in two alternative ways suggestive of two different conformations, ‘intermediate’ and ‘closed’ (Supplementary information, Figure S3). In the ‘intermediate’ conformation, the guanidinium group of Arg23 is 4.36 Å away from the hydroxymethyl group (Figure 3B and E), whereas in the ‘closed’ conformation, the guanidinium group is 2.73 Å away from the hydroxymethyl group and 2.37 Å away from the phosphate group of hmCMP (Figure 3C and F), forming hydrogen bonds with them. As the positive charge of the guanidinium group is delocalized, it enables the formation of multiple hydrogen bonds with its binding partner. The ‘intermediate’ and ‘closed’ conformation might arise from alternative hydrogen bonding to each of the three positively charged nitrogen atoms of the guanidinium group. When hmCMP is bound to MilB, the guanidinium group of Arg23 is induced to rotate 65° toward the direction of the 5-hydroxymethyl group of hmCMP, leading to the formation of the ‘closed’ conformation of the binding pocket. The stacking interaction between the benzene ring of Phe22 and the pyrimidine ring of the 5hmC moiety also contributes to the stabilization of the binding of hmCMP to the active site pocket of MilB.

In BcmB and BlsM, the equivalent positions to Arg23 in MilB are replaced by a lysine residue (Lys12) and a methionine residue (Met25), respectively (Figure 5). Like MilB, BcmB has a substrate specificity biased toward hmCMP, which can be rationalized by the similar positive charge and side chain length between arginine and lysine residues. By contrast, BlsM exhibits higher specificity for CMP (25) compared with hmCMP (Table 1 and Supplementary information, Figure S7D). The shorter and uncharged side chain of methionine at this position might be incapable of specifically recognizing hmCMP and discriminating it from CMP.

In order to test whether changing arginine to methionine is sufficient to reverse the substrate preference, MilB-R23M was constructed. WT MilB preferentially hydrolyzes hmCMP, and its hydrolyzing efficiency toward CMP is 142-fold lower (Table 1). In contrast, the R23M mutation of MilB resulted in a four-fold decrease in its hydrolysis activity for hmCMP and a five-fold increase in its hydrolysis activity for CMP, corresponding to a 20-fold inversion of the substrate specificity (Table 1). However, there are variations in the kinetic parameters of MilB for hmCMP between this study and that reported in reference (8), such as *K*_M_ differs by 68.45 μM, and *k*_cat_ differs by 50.03 × 10^−3^ s^−1^. As the *k*_cat_ of MilB for hmCMP in reference (8) is 3.1×10^−3^ s^−1^, even lower than that for MilB for CMP reported by Megan D. Sikowitz *et al.* (9), the kinetic data obtained by this study, therefore, look more consistent and convincing (Table 1). These variations might arise by the way for calculating the peak area of the products, type of column and separation conditions used for HPLC as well as protein activity and purity in different preparations.

### The conserved phenylalanine and arginine residues in the recognition of 5mC and 5hmC

The structure of NgTet1 in complex with 5-methylcytosine DNA revealed that NgTet1 uses a base-flipping mechanism to access 5mC. The flipped 5mC is positioned in the active site pocket with planar stacking contacts specific for 5mC. The extra-helical 5mC is bound in a cage-like active site via stacking of the flipped base in between Phe295 and the guanidinium group of Arg224 (Supplementary information, Figure S5A).

Similarly, in the structure of MilB in complex with hmCMP, there are also a conserved Phe22 and a conserved Arg23 at the 5hmC-binding site (Supplementary information, Figure S5B). Sequence alignment of MilB, BcmB, BlsM, as well as other putative MilB homologs found by the COBALT searching tool (http://www.ncbi.nlm.nih.gov/tools/cobalt/cobalt.cgi) indicate that they all possess the conserved ‘F-L-(G/A)-(G/A)-P-F-X-X-L-(L/V/T)’ motif (X represents any residue; Figure 5). In another 5mC-binding protein, the pyrimidine ring of 5mC was stabilized by the planar stacking of two aromatic amino acids Y471 and Y483 (12). We here propose that the ‘F-L-(G/A)-(G/A)-P-F-X-X-L-(L/V/T)’ motif is crucial for the recognition of the pyrimidine ring of cytosine, while the ‘F-L-(G/A)-(G/A)-P-F-(R/K)-X-L-(L/V/T)’ motif is specific for recognizing hydroxymethyl CMP. A good case to support this hypothesis is BlsM. BlsM has no special preference for hmCMP or CMP, and has a motif of ‘F-L-X-X-P-F-M-X-L-(L/V/T)’ at its active site. Interestingly, genes of MilB homologs with the ‘F-L-(G/A)-(G/A)-P-F-(R/K)-X-L-(L/V/T)’ motif are genetically linked with putative homologs of MilA, which is a CMP hydroxymethylase, whereas this kind of genomic organization does not exist in MilB homologs such as BlsM, which do not have the conserved Arg/Lys residue in the ‘F-L-(G/A)-(G/A)-P-F-X-X-L-(L/V/T)’ motif.

### A proposed model for how MilB specifically recognizes hmCMP and catalyzes the hydrolysis of its *N*-glycosidic bond

Based on our structural, enzymatic and bioinformatic results, we propose a functional model for how MilB specifically recognizes hmCMP and catalyzes its *N*-glycosidic bond hydrolysis. When the substrate hmCMP is not bound to MilB, the conformation of Arg23 exists in the ‘open’ state (Figure A). When hmCMP is recognized by the active site of MilB, its phosphate and 5-hydroxymethyl groups form hydrogen bonds with the Arg23 residue of MilB, bending its guanidinium group backward toward the active site pocket to form the ‘closed’ state, poised for the cleavage of the *N*-glycosidic bond of hmCMP (Figure B). After hydrolysis of the *N*-glycosidic bond, the ribose 5′-phosphate and 5hmC molecules are separated due to thermal motion. Neither of them has sufficient binding affinity for Arg23; therefore, Arg23 tends to relax to the stretched conformation (Figure C). Finally, the products of the hydrolysis reaction, ribose 5′-phosphate and 5hmC, are released from the active site pocket, and Arg23 returns to the ‘open’ state (Figure D).8

**Figure 8.**
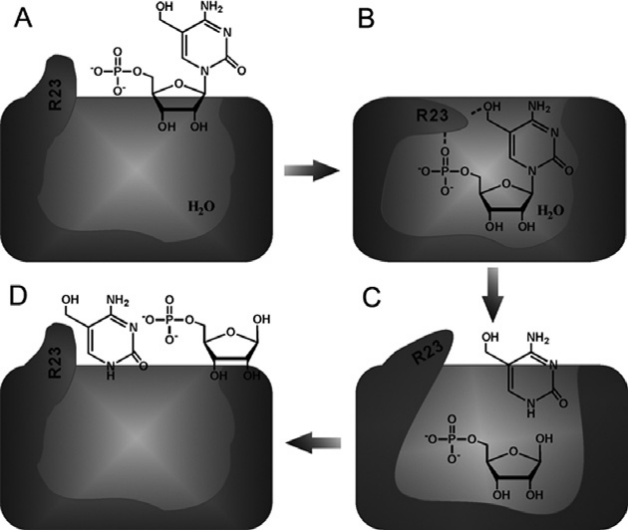
Proposed model for how MilBrecognizes hmCMP and catalyzes its hydrolysis, in which Arg23 functions as a gate to control the substrate entry and products release. (A) When the substrate hmCMP is not bound to MilB, the conformation of Arg23 exists in the “open” state. (B) Upon the recognition of hmCMP by the active site of MilB, its phosphate and 5-hydroxymethyl groups form hydrogen bonds with the Arg23 residue of MilB, bending its guanidinium group backward towards the active site pocket to form the “closed” state. The MilB enzyme is thus poised for the cleavage of the N-glycosidic bond of hmCMP. (C) After hydrolysis of the glycosidic bond, the reaction products, ribose 5’-phosphate and 5hmC, are separated due to thermal motion. Neither of them has sufficient affinity for Arg23, therefore Arg23 tends to relax to the stretched conformation. (D) The products of the hydrolysis reaction, ribose 5’-phosphate and 5hmC, are released. Arg23 of MilB returns to the “open” state.

In this way, Arg23 of MilB functions as a gate of the ‘magic hydrolysis cage’ of MilB to regulate the substrate entry and products release. When this gate is open, it specifically recognizes the hmCMP substrate and permits its entry into the ‘hydrolysis cage.’ This gate is then locked to the ‘closed’ state by hmCMP, which comes into the ‘hydrolysis cage.’ After the hydrolysis reaction inside the ‘magic cage’ is over, this gate is opened up again to release the shredded fragments. This hypothesized versatility of Arg23 is also supported by our normal mode analysis result, which shows that it is located on a highly mobile loop undergoing constant movement (Supplementary information, Video S1).

## SUPPLEMENTARY DATA

Supplementary Data are available at NAR Online.

SUPPLEMENTARY DATA
