# Supplementary material for: Structure of the N-glycosidase MilB in complex with hydroxymethyl CMP reveals its Arg23 specifically recognizes the substrate and controls its entry
Source: Nucleic Acids Res. 2014 Jun 11;42(12):8115–24. doi: 10.1093/nar/gku486 (PMC4081090; doi:10.1093/nar/gku486)
Supplement: SUPPLEMENTARY DATA [file supp_42_12_8115__index.html]

Structure of the N-glycosidase MilB in complex with hydroxymethyl CMP reveals its Arg23 specifically recognizes the substrate and controls its entry — SUPPLEMENTARY DATA 

# Structure of the *N*-glycosidase MilB in complex with hydroxymethyl CMP reveals its Arg23 specifically recognizes the substrate and controls its entry

## SUPPLEMENTARY DATA

**Files in this Data Supplement:**

- Supplementary Figures and Table
- Supplementary Vedio
