# Supplementary material for: Structure of the N-glycosidase MilB in complex with hydroxymethyl CMP reveals its Arg23 specifically recognizes the substrate and controls its entry
Source: Nucleic Acids Res. 2014 Jun 11;42(12):8115–24. doi: 10.1093/nar/gku486 (PMC4081090; doi:10.1093/nar/gku486)
Supplement: SUPPLEMENTARY DATA [file supp_gku486_nar-00564-m-2014-File010.doc]

**Supplementary information, Table S1** Plasmids used in this study.

| **Plasmids** | **Relevant properties** | **Source of reference** |
| --- | --- | --- |
| pJTU2956 | expression vector for MilB, which was cloned into the NdeI and BamHI restriction sites of pET28a(+) | [8] |
| pET41BlsM | BlsM expression vector | [17] |
| pJTU4391 | MilB-E103A expression vector | This study |
| pJTU4392 | MilB-R23M expression vector | This study |
| pJTU4393 | MilB-R23A expression vector | This study |
| pJTU4394 | MilB-R23E expression vector | This study |
| pJTU4395 | MilB-R23K expression vector | This study |
| pJTU4396 | MilB-R23L expression vector | This study |
| pJTU4397 | MilB-R23S expression vector | This study |

**Supplementary information, Table S2** Primers used in this study.

|  | **Forward primer** | **Reverse primer** |
| --- | --- | --- |
| MilB-E103A | cccggcacccatgtggcgatcggctgggcgagc | gctcgcccagccgatcgccacatgggtgccggg |
| MilB-R23M | gggccgttcATGcagctcgtcgacccccg | gacgagctgCATgaacggcccgccgaggaag |
| MilB-R23A | gggccgttcGCCcagctcgtcgacccccg | gacgagctgGGCgaacggcccgccgagg |
| MilB-R23E | gggccgttcGAAcagctcgtcgacccccg | gacgagctgTTCgaacggcccgccgagg |
| MilB-R23K | gggccgttcAAAcagctcgtcgacccccg | gacgagctgTTTgaacggcccgccgagg |
| MilB-R23L | gggccgttcCTGcagctcgtcgacccccg | gacgagctgCAGgaacggcccgccgagg |
| MilB-R23S | gggccgttcAGCcagctcgtcgacccccg | gacgagctgGCTgaacggcccgccgagg |

**Supplementary information, Table S3** Data collection and refinement statistics.

|  | SeMet-MilB | MilB | The MilB-E103A/hmCMP complex |
| --- | --- | --- | --- |
| **Data collection** |  |  |  |
| Space group | *C*222_1_ | *C*222_1_ | *C*222_1_ |
| Unit cell parameters |  |  |  |
| *a*, *b*, *c* (Å) | 45.9, 102.1, 81.7 | 46.3, 102.0, 82.0 | 45.4, 101.8, 81.7 |
| α, β, γ (°) | 90, 90, 90 | 90, 90, 90 | 90, 90, 90 |
| Resolution (Å) | 50-2.25 (2.33-2.25) | 50-1.80 (1.86-1.80) | 50-2.40 (2.49-2.40) |
| *R*_merge_ (%) | 14.3 (50.3) | 10.4 (28.7) | 12.5 (46.0) |
| *I* /σ*I* | 12.6 (3.1) | 13.9 (8.9) | 9.2 (3.4) |
| Completeness (%) | 98.4 (93.6) | 96.4 (99.9) | 92.1 (98.7) |
| Redundancy | 13.3 (10.4) | 12.3 (14.4) | 4.4 (4.2) |
| **Refinement** |  |  |  |
| Resolution (Å) | 50-2.25 | 50-1.80 | 50-2.40 |
| Number of reflections | 122,549 | 219,378 | 31,144 |
| *R*_work_ / *R*_free_ (%) |  | 18.3/21.6 | 19.2/25.7 |
| Number of atoms |  |  |  |
| Protein |  | 1,202 | 1,217 |
| Ligand/ion |  | 0 | 23 |
| Water |  | 183 | 38 |
| *B*-factors (Å^2^) |  |  |  |
| Overall |  | 19.8 | 41.3 |
| Protein |  | 17.6 | 41.6 |
| Ligand/ion |  | N/A | 35.5 |
| Water |  | 34.4 | 34.6 |
| RMSD Bond length (Å) |  | 0.006 | 0.015 |
| RMSD Bond angles (°) |  | 1.151 | 1.761 |

Data for each structure were collected or calculated from a single crystal. RMSD, root-mean-square deviations from the ideal geometry. Data for the highest resolution shell are shown in parentheses.

**
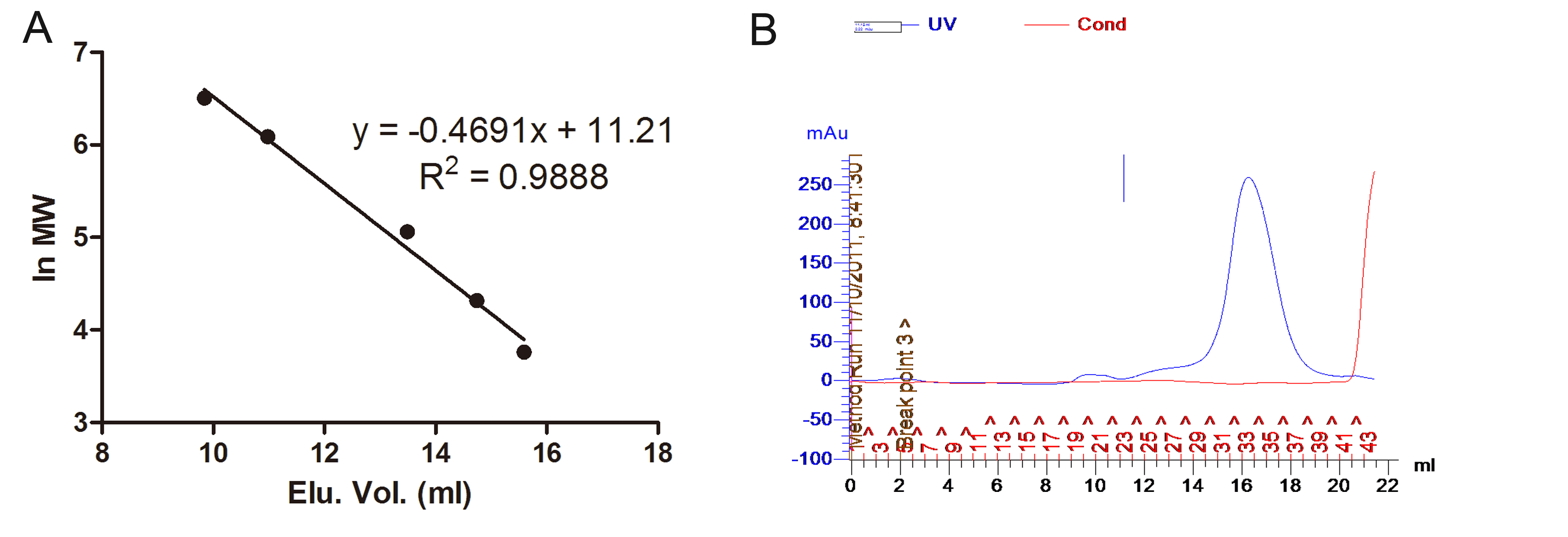
**

**Supplementary information, Figure S1 (A)** The standard calibration curve of the Superdex200 size exclusion chromatography. **(B)** The Superdex200 size exclusion chromatography elution profile of MilB. The elution volume of MilB is 16.2 mL. Compared with the standard curve in (A), the molecular weight of MilB is about 40 kDa, indicating that MilB exists as a dimer in solution.

**
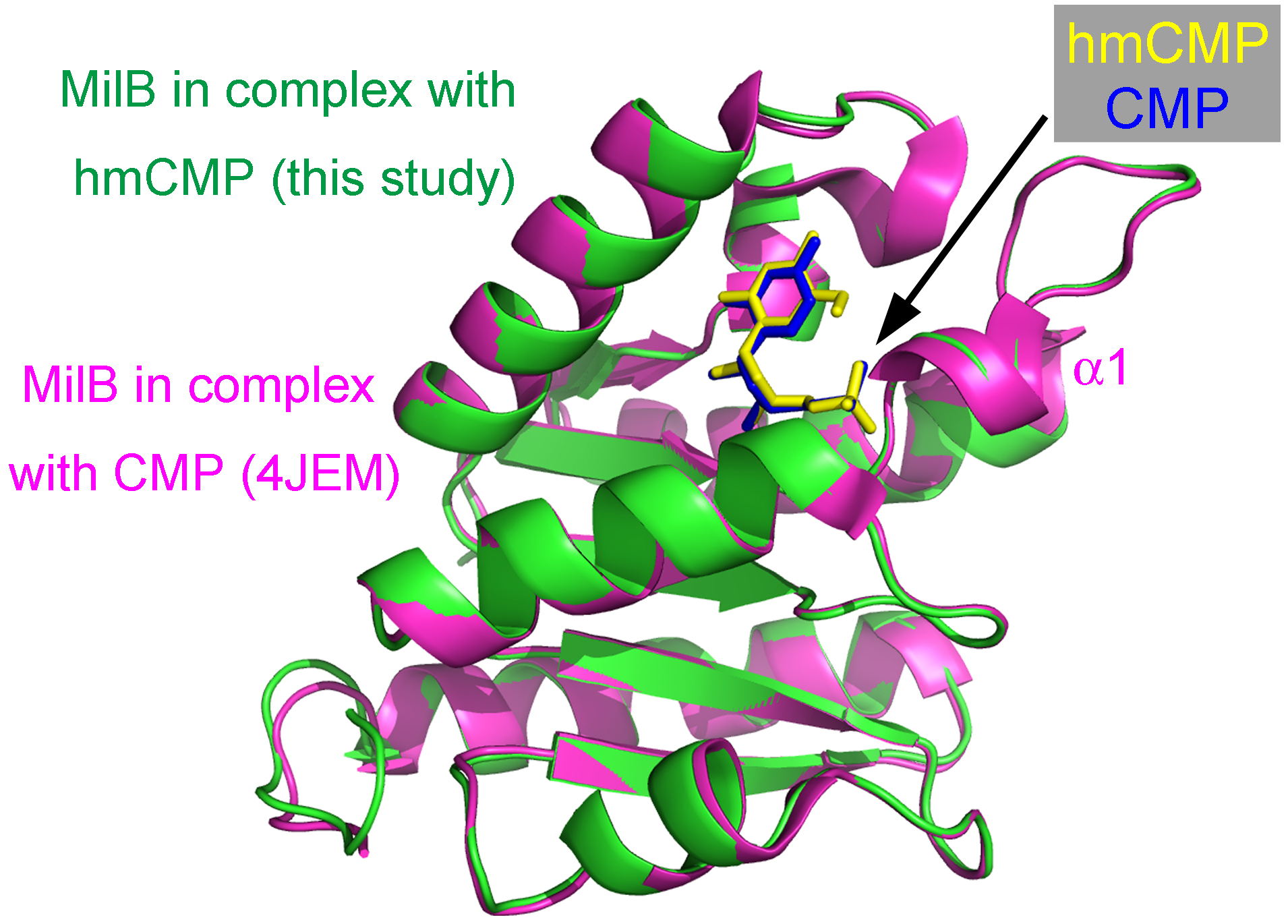
**

**Supplementary information, Figure S2** Comparison of the crystal structure of MilB in complex with hmCMP determined in this study and that of MilB in complex with CMP (PDB code: 4JEM) reported previously [9]. In the structure of MilB in complex with hmCMP, MilB is colored in green and hmCMP is colored in yellow. In the structure of MilB in complex with CMP, MilB is colored in magenta and CMP is colored in blue. Helix α1, where Arg23 resides, in the structure of MilB in complex with CMP is indicated.


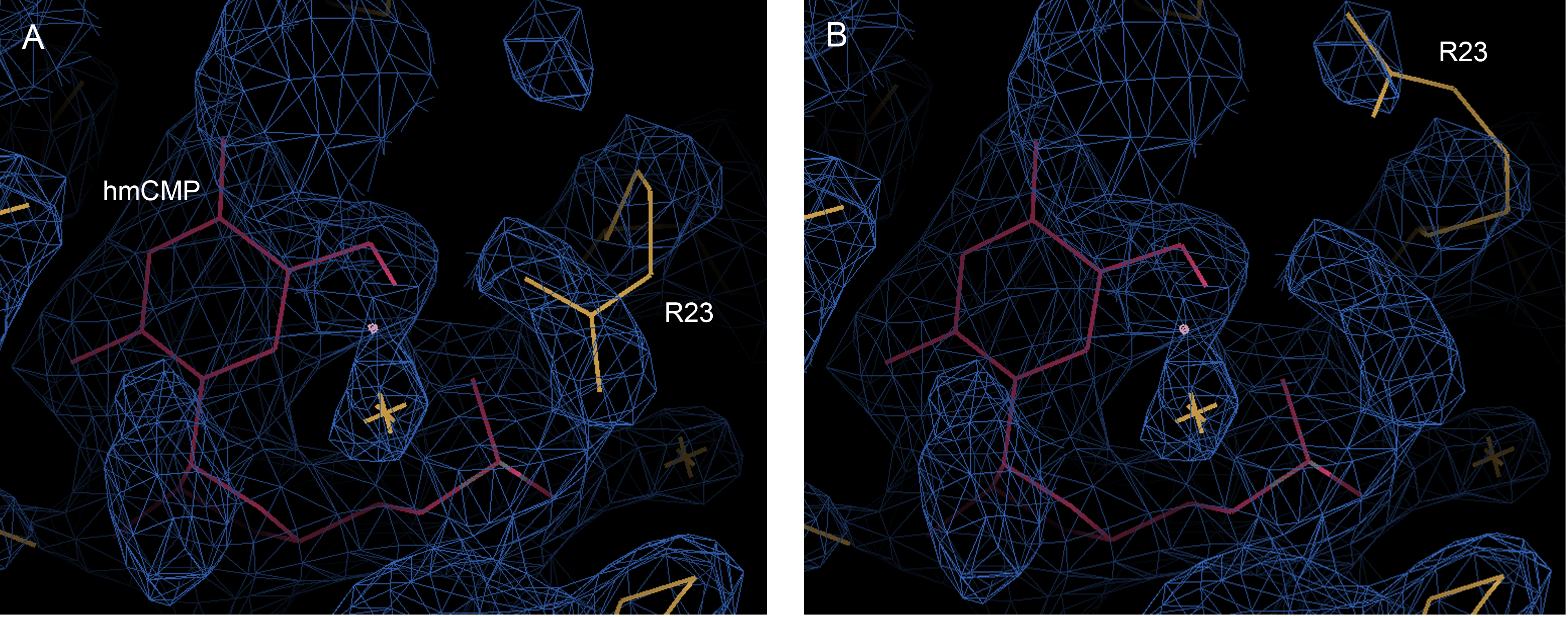


**Supplementary information, Figure S3** The 2Fo - Fc electron density map around Arg23 of MilB and the bound hmCMP. The map is of the crystal structure of the MilB-E103A/hmCMP complex, and is contoured at the 1σ contour level (blue). Models of MilB and hmCMP are colored in yellow and magenta, respectively. The side chain of Arg23 can be placed in two alternative ways, suggestive of two different conformations. **(A)** In the “closed” conformation, Arg23 is completely bended to interact with hmCMP. **(B)** In the “intermediate” state, the side chain of Arg23 exists in a half-bended-half-stretched conformation.


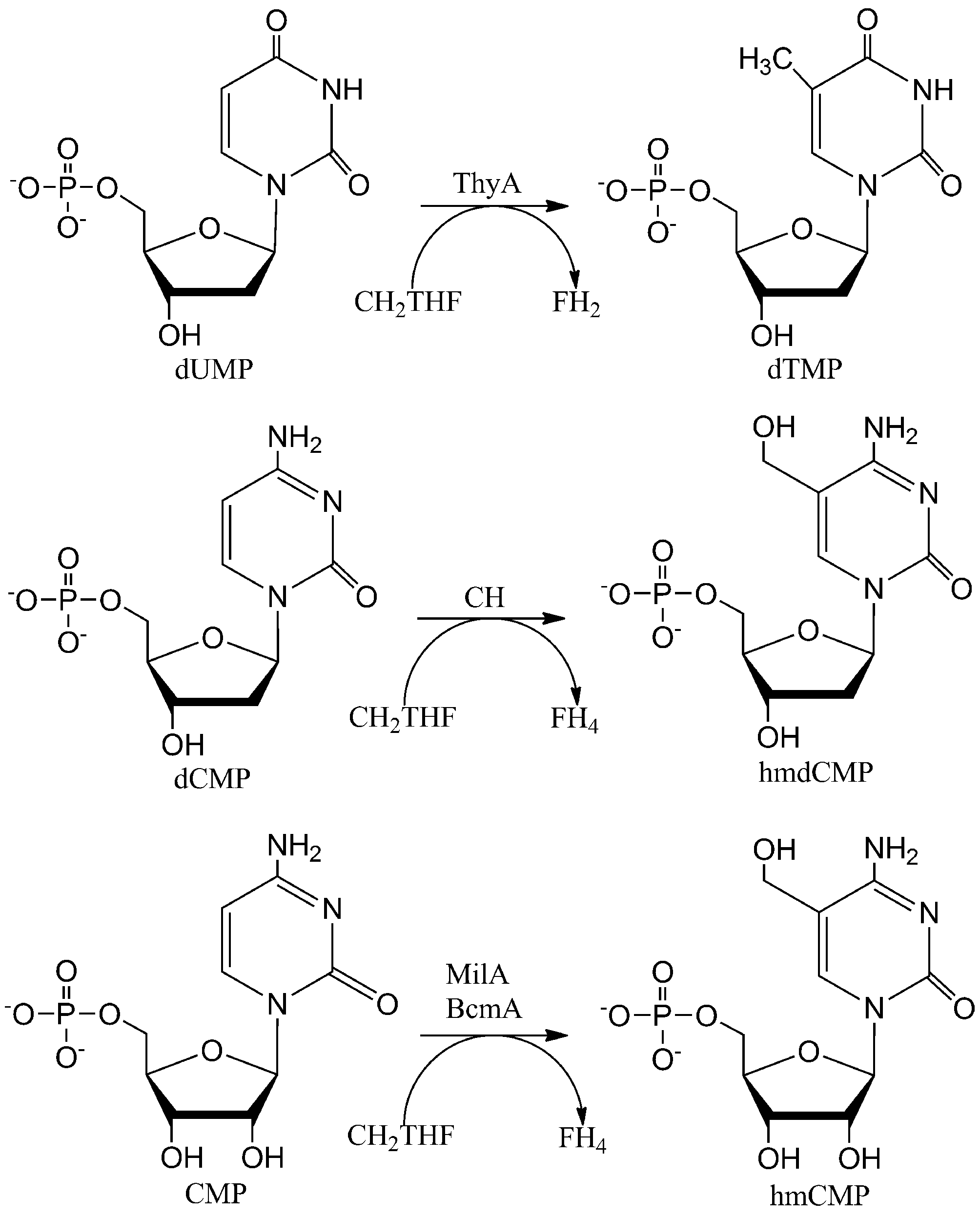


**Supplementary information, Figure S4** The reaction catalyzed by MilA and its homologs. MilA homologs are annotated either as thymidylate synthase A (ThyA) or deoxycytidylate hydroxymethylase (CH) in the database. ThyA catalyzes the C5-methylation of dUMP to form dTMP, whereas CH first methylates dCMP and then hydroxylates the methyl group to form hmdCMP. MilA and BcmA catalyze the hydroxymethylation of CMP to form hmCMP. The donor of the methyl group for the above three types of reaction is 5,10-methylenetetrahydrofolate (CH_2_THF).


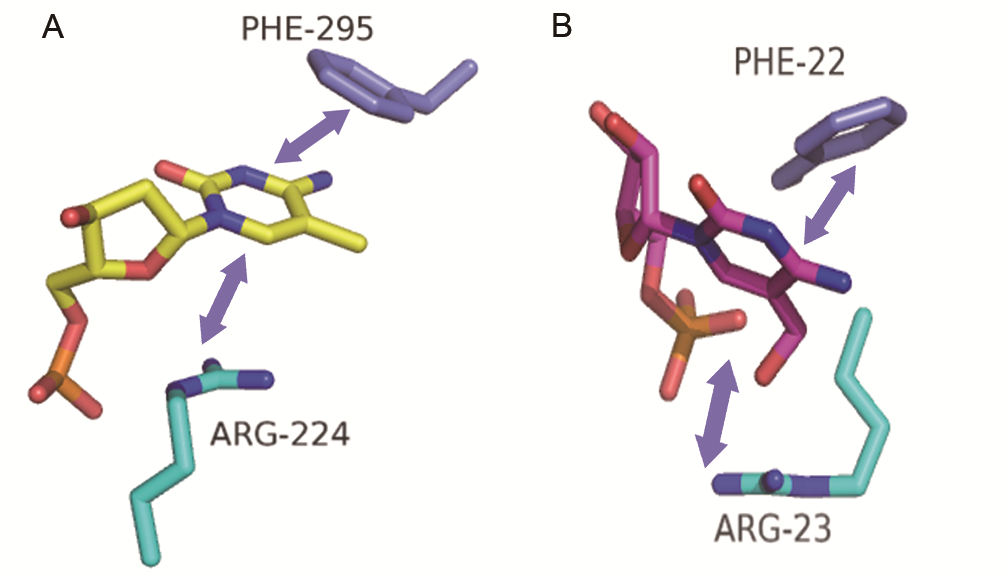


**Supplementary information, Figure S5** The similar spatial organizations of phenylalanine and arginine residues in the recognition of 5mC or 5hmC. **(A)** Phe295 in the structure of NgTet1 in complex with 5-methylcytosine DNA form planar π stacking contacts with the extra-helical 5mC. Arg224 is also important for the binding. **(B)** In the structure of MilB in complex with hmCMP, the benzene ring of the conserved Phe22 and the guanidinium group of Arg23 are crucial for the association with the 5hmC moiety of hmCMP.

**
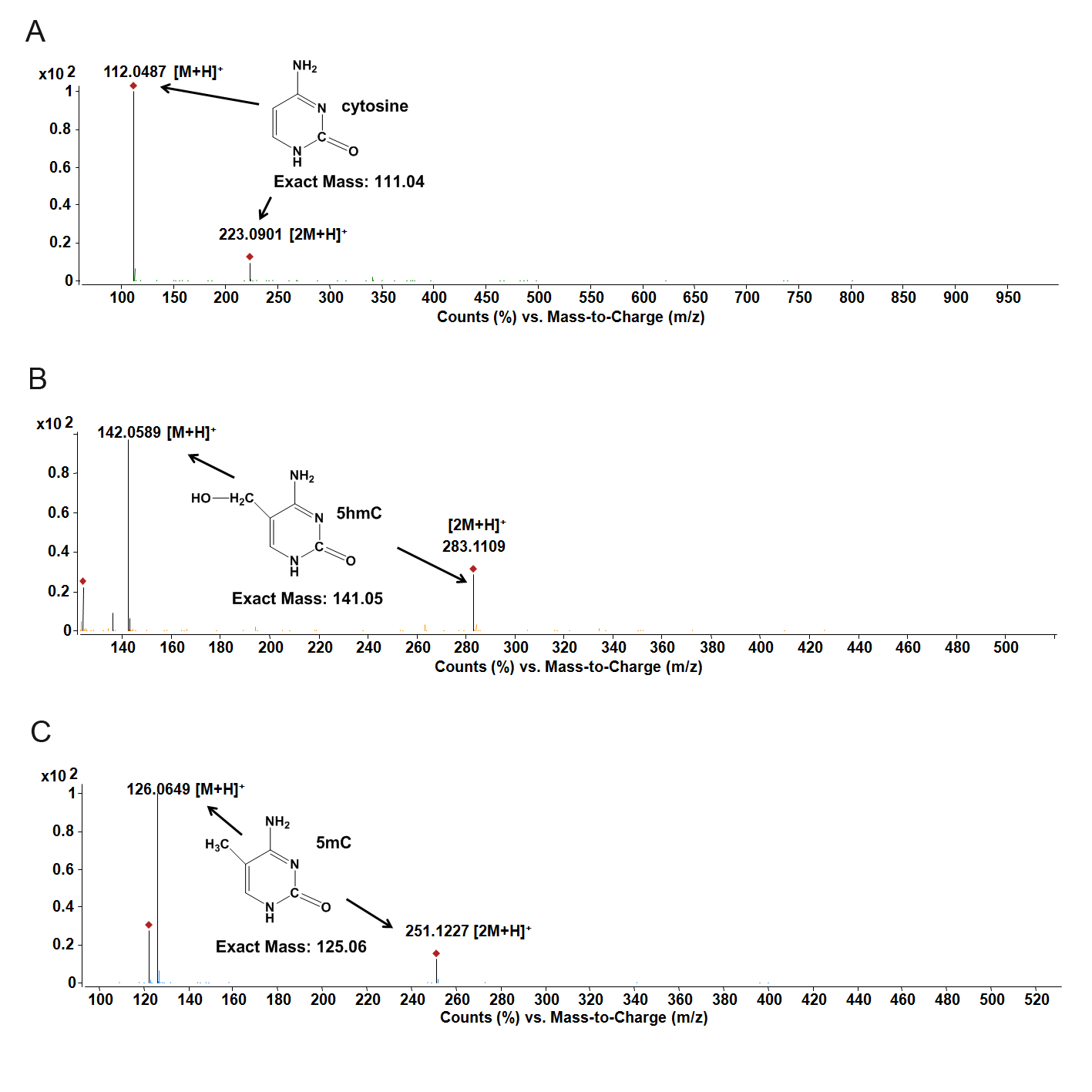
**

**Supplementary information, Figure S6** Q-TOF/MS analysis of cytosine, 5hmC and 5mC produced by the hydrolysis reaction of MilB/MilB-R23M with CMP/5hmCMP/5mCMP. (**A**) Determination of the peak in LC corresponds to cytosine by Q-TOF/MS. The theoretical mass of cytosine is 111.04, while the [M+H]^+^ and [2M+H]^+^ of cytosine produced by the reaction of MilB/MilB-R23M with CMP were analyzed as 112.0478 and 223.0901. (**B**) Determination of the peak in LC corresponds to 5-hydroxymethylcytosine by Q-TOF/MS.The theoretical mass of 5hmC is 141.05, while the [M+H]^+^ and [2M+H]^+^ of 5hmC produced by the reaction of MilB/MilB-R23M with 5hmCMP were analyzed as 142.0589 and 283.1109. (**C**) Determination of the peak in LC corresponds to 5-methylcytosine by Q-TOF/MS.The theoretical mass of 5mC is 125.06, while the [M+H]^+^ and [2M+H]^+^ of 5mC produced by the reaction of MilB/MilB-R23M with 5mCMP were analyzed as 126.0649 and 251.1227.

**
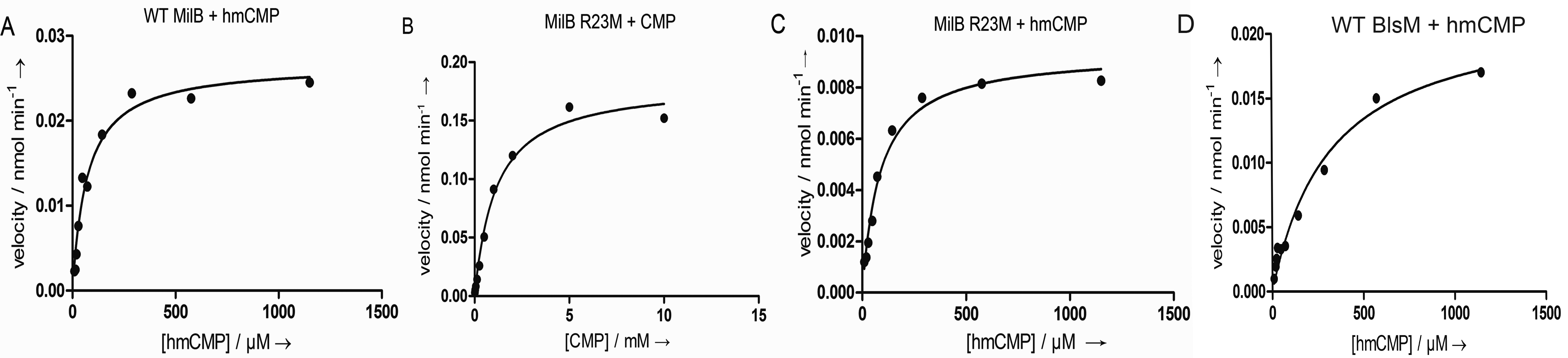
**

**Supplementary information, Figure S7** Measurement of the enzymatic kinetic constants of WT MilB and MilB-R23M, using hmCMP or CMP as the substrate. (**A**) 5hmC formation catalyzed by WT MilB, with hmCMP as the substrate. (**B**) Cytosine formation catalyzed by MilB-R23M, with CMP as the substrate. (**C**) 5hmC formation catalyzed by MilB-R23M, with hmCMP as the substrate. (**D**) 5hmC formation catalyzed by BlsM, with hmCMP as the substrate.

**
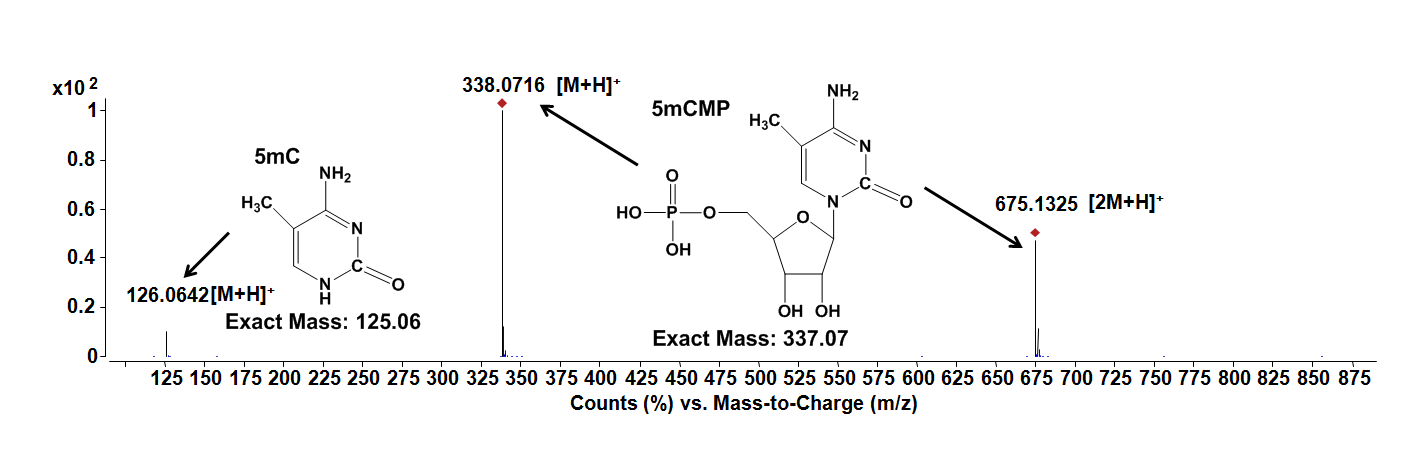
**

**Supplementary information, Figure S8** Q-TOF MS analysis of 5mCMP. The theoretical mass of 5mCMP is 337.07, while the [M+H]^+^ and [2M+H]^+^ of 5mCMP were analyzed as 338.0716 and 675.1325. The ion 126.0642 with low abundance corresponds to mooeity of 5-methylcytosine that is formed during the Q-TOF/MS fragmentation.

**Supplementary information, Video S1** Normal mode analysis of MilB (colored in green). The side chain of the crucial Arg23 residue is shown in stick representation, with its carbon and nitrogen atoms colored in yellow and blue, respectively.
