# Supplementary figures and images for: Structure of the N-glycosidase MilB in complex with hydroxymethyl CMP reveals its Arg23 specifically recognizes the substrate and controls its entry
Source: Nucleic Acids Res. 2014 Jun 11;42(12):8115–24. doi: 10.1093/nar/gku486 (PMC4081090; doi:10.1093/nar/gku486)

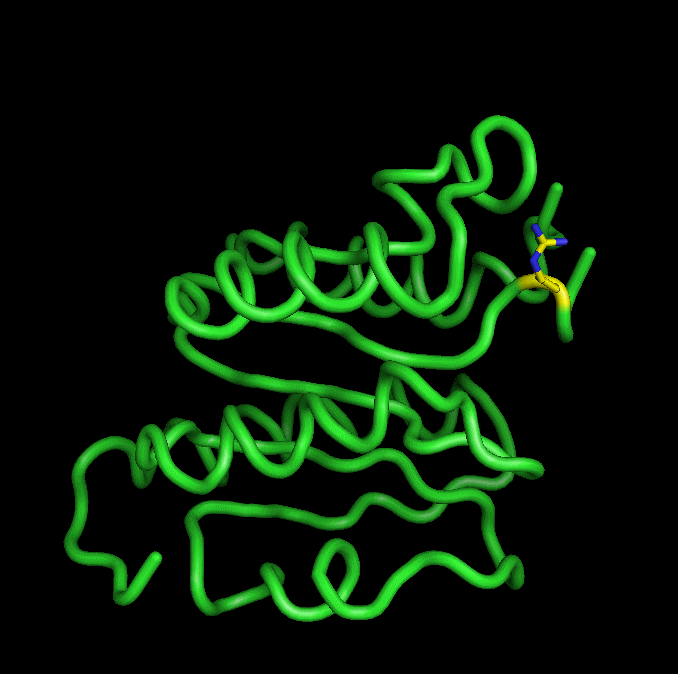

Supplement: SUPPLEMENTARY DATA [file supp_gku486_nar-00564-m-2014-File011.zip › Supplementary vedio S1.gif]
